# Determinants of implementation for group medical visits for patients with chronic pain: a systematic review

**DOI:** 10.1186/s43058-024-00595-8

**Published:** 2024-05-23

**Authors:** Isabel Roth, Malik Tiedt, Carrie Brintz, Ariana Thompson-Lastad, Gayla Ferguson, Erum Agha, Jennifer Holcomb, Paula Gardiner, Jennifer Leeman

**Affiliations:** 1 Department of Physical Medicine and Rehabilitation, Program on Integrative Medicine, University of North Carolina at Chapel Hill School of Medicine, Chapel Hill, NC USA; 2 Department of Health Studies and Applied Educational Psychology, Program in Nutrition, Teachers College, Columbia University, New York, NY USA; 3 Department of Anesthesiology, Division of Pain Medicine, Vanderbilt University Medical Center, Nashville, TN USA; 4 Center for Musculoskeletal Research, Vanderbilt University Medical Center, Nashville, TN USA; 5 Osher Center for Integrative Health, Vanderbilt University Medical Center, Nashville, TN USA; 6 Department of Family and Community Medicine, Osher Center for Integrative Health, University of California San Francisco, San Francisco, CA USA; 7 Department of Management, Policy, and Community Health, University of Texas Health Science Center at Houston School of Public Health, Houston, TX USA; 8 Department of Psychiatry, University of North Carolina at Chapel Hill School of Medicine, Chapel Hill, NC USA; 9 Sinai Chicago, Sinai Urban Health Institute, Chicago, IL USA; 10 Department of Family Medicine, Cambridge Health Alliance, University of Massachusetts Medical School, Boston, MA USA; 11 University of North Carolina at Chapel Hill School of Nursing, Chapel Hill, NC USA

## Abstract

**Background:**

Despite the critical need for comprehensive and effective chronic pain care, delivery of such care remains challenging. Group medical visits (GMVs) offer an innovative and efficient model for providing comprehensive care for patients with chronic pain. The purpose of this systematic review was to identify barriers and facilitators (determinants) to implementing GMVs for adult patients with chronic pain.

**Methods:**

The review included peer-reviewed studies reporting findings on implementation of GMVs for chronic pain, inclusive of all study designs. Pubmed, EMBASE, Web of Science, and Cochrane Library were searched. Studies of individual appointments or group therapy were excluded. The Mixed Methods Appraisal Tool was used to determine risk of bias. Data related to implementation determinants were extracted independently by two reviewers. Data synthesis was guided by the updated Consolidated Framework for Implementation Research.

**Results:**

Thirty-three articles reporting on 25 studies met criteria for inclusion and included qualitative observational (*n* = 8), randomized controlled trial (*n* = 6), quantitative non-randomized (*n* = 9), quantitative descriptive (*n* = 3), and mixed methods designs (*n* = 7). The studies included in this review included a total of 2364 participants. Quality ratings were mixed, with qualitative articles receiving the highest quality ratings. Common multi-level determinants included the relative advantage of GMVs for chronic pain over other available models, the capability and motivation of clinicians, the cost of GMVs to patients and the health system, the need and opportunity of patients, the availability of resources and relational connections supporting recruitment and referral to GMVs within the clinic setting, and financing and policies within the outer setting.

**Conclusions:**

Multi-level factors determine the implementation of GMVs for chronic pain. Future research is needed to investigate these determinants more thoroughly and to develop and test implementation strategies addressing these determinants to promote the scale-up of GMVs for patients with chronic pain.

**Trial registration:**

This systematic review was registered with PROSPERO 2021 CRD42021231310.

**Supplementary Information:**

The online version contains supplementary material available at 10.1186/s43058-024-00595-8.

Contributions to the literature

- Chronic pain impacts about a fifth of adults in the United States, but access to gold-standard pain management care remains limited.
- Group medical visits (GMVs) are an innovation that may help to provide access to gold-standard pain management at scale, but uptake has been limited.
- This is the first systematic review to investigate barriers and facilitators to GMVs for chronic pain specifically. The determinants found in the studies in this review suggest that implementation strategies should target determinants at multiple levels.
- Future research is needed on both implementation determinants and strategies for GMVs for chronic pain.

## Background

Chronic pain is estimated to affect at least 20% of adults in the United States, with 7% of adults suffering from high-impact chronic pain that hinders their daily life and activities [1]. The societal toll of chronic pain is immense, contributing not only to large economic costs, but also to the suffering of individuals, families and communities [2, 3]. Chronic pain is also associated with significant comorbidities, opioid abuse, and poorer overall health. Groups with low socioeconomic status and racial/ethnic minorities are disproportionately affected by and undertreated for chronic pain [4–8]. The latest guidelines for chronic pain management recommend a biopsychosocial approach to care that is multi- or interdisciplinary, includes evidence-based nonpharmacological approaches, pain self-management support, and a trusting patient-clinician relationship [3, 9–13]. Despite the critical need for comprehensive and effective chronic pain care, the delivery of such care remains challenging. Comprehensive nonpharmacological approaches to pain management are resource intensive, involving multiple visits over time [3, 10–12, 14, 15].

Group medical visits (GMVs) offer an innovative and efficient model for providing comprehensive care for patients with chronic pain [16]. ‘Group medical visit’ and ‘shared medical appointment’ are broad terms used to describe multiple models of care that include a) care from one or more licensed clinicians, b) peer support, and c) health education. GMVs have the potential to meet the goals of the Quintuple Aim for healthcare quality improvement by 1) improving patient experiences (extended time with the provider, peer support, and engagement in care); 2) improving population health (improved pain managemenet); 3) lowering health care costs (more efficient care delivery); 4) improving provider experience, (increased job satisfaction); and 5) improving health equity (increasing access to guideline-concordant pain care for underserved communities) [17–26]. To achieve these goals, GMVs for treatment of chronic pain need to be more widely implemented and sustained.

Previous systematic reviews of GMVs have not focused specifically on chronic pain alone or looked specially at determinants of implementation for this innovation [27–31]. In this systematic review, we explored how GMVs for patients with chronic pain are implemented into clinical settings and identified factors that may determine when implementation is or is not successful. We utilized the updated Consolidated Framework for Implementation Research (CFIR), a widely-used implementation framework, to provide structure in identifying factors that determine successful implementation of innovations in multiple domains [32].

The goal of this review is to set the stage for development of implementation strategies to promote the widespread uptake of GMVs for chronic pain into clinical care. As well as informing clinical practice, the findings from this review may help identify important directions for future implementation research.

## Methods

### Study design

This mixed methods systematic review followed PRISMA guidelines, which are considered the gold standard for reporting [33, 34]. A review protocol in accordance with the PROSPERO guidelines for systematic reviews outlined the procedure to be adhered to during the review [35]. The protocol ensured that appropriate databases, key words and search terms were included. Experts in GMVs and implementation science reviewed the relevance of the search terms. The final systematic review protocol was established thorough an iterative process and was submitted to PROSPERO for registration (PROSPERO 2021 CRD42021231310) [36].

### Search methods

The review included peer-reviewed studies that reported findings on the implementation of GMVs for chronic pain. Pubmed, EMBASE, Web of Science, and Cochrane Library were searched on October 10th, 2022. Search terms included “chronic pain,” “fibromyalgia,” “diabetic neuropathies,” “low back pain,” “headache disorders, “sickle cell anemia,” “arthritis,” “neurogenic pain” and “shared medical appointments” or “group medical visit,” as well as permutations of all terms using Boolean logic. For a detailed search strategy, see Appendix A.

Quantitative, qualitative, and mixed methods study designs, inclusive of experimental and observational study designs, were included. Opinion papers, protocol papers, systematic reviews (and other reviews such as scoping or narrative reviews), and editorials were excluded. Only full-length publications were included; conference abstracts were excluded. English language studies regardless of country where research was conducted were included.

Studies of GMVs (alternatively referred to as shared medical appointments or medical group visits) that focused on management of chronic pain conditions were included. For the purposes of this review, chronic pain is defined as pain that lasts more than three months.

GMVs are defined for the purposes of this review as:

- Care is provided to multiple patients in the same room or telehealth meeting.
- A licensed clinician documents the medical encounter (provider bills insurance using relevant ICD-10 codes and documents in the medical record).
- Patients interact with each other during the group session.

Studies of individual medical appointments (not groups) for chronic pain were excluded. Studies of group therapy where no medical codes were billed and no medical provider was present were excluded, as group therapy with no medical component is a distinct intervention from GMVs.

### Data extraction and synthesis

Two reviewers independently screened titles and abstracts using the inclusion and exclusion criteria. When disagreement occurred, two reviewers independently assessed full texts and came to an agreement.

Two reviewers extracted data from each article using a structured tool to extract key features of the included studies related to method, sampling approach, sample size, and characteristics of the study sample, innovation, and setting. Data related to implementation determinants (i.e., barriers and facilitators) were extracted from throughout the body of the manuscripts, as relevant information on contextual factors influencing implementation may be included throughout the body of the text. The data source and context within the text were extracted for each determinant. Reviewers compared extractions and reconciled differences.

Data synthesis was guided by the Consolidated Framework for Implementation Research (CFIR) [32], a comprehensive framework of determinants related to implementation. CFIR contains five domains (Innovation Characteristics, Inner Setting, Outer Setting, Characteristics of Individuals, and Process) and constructs within each domain. Determinants data were compiled and grouped by category by two reviewers independently and then sorted into CFIR domains.

To assess the quality of each article, two reviewers independently extracted data and assessed risk of bias using the Mixed Methods Appraisal Tool version 2018 [37]. Quality was assessed to determine the risk of bias in the findings presented in the included manuscripts. The Mixed Methods Appraisal Tool allowed for the assessment of bias across a broad range of study types. The tool provides distinct checklists of criteria to evaluate qualitative, quantitative, or mixed methods studies. Discrepancies were resolved by referral to the original studies and occasionally through arbitration by a third reviewer.

## Results

### Description of included studies

**Fig. 1:**
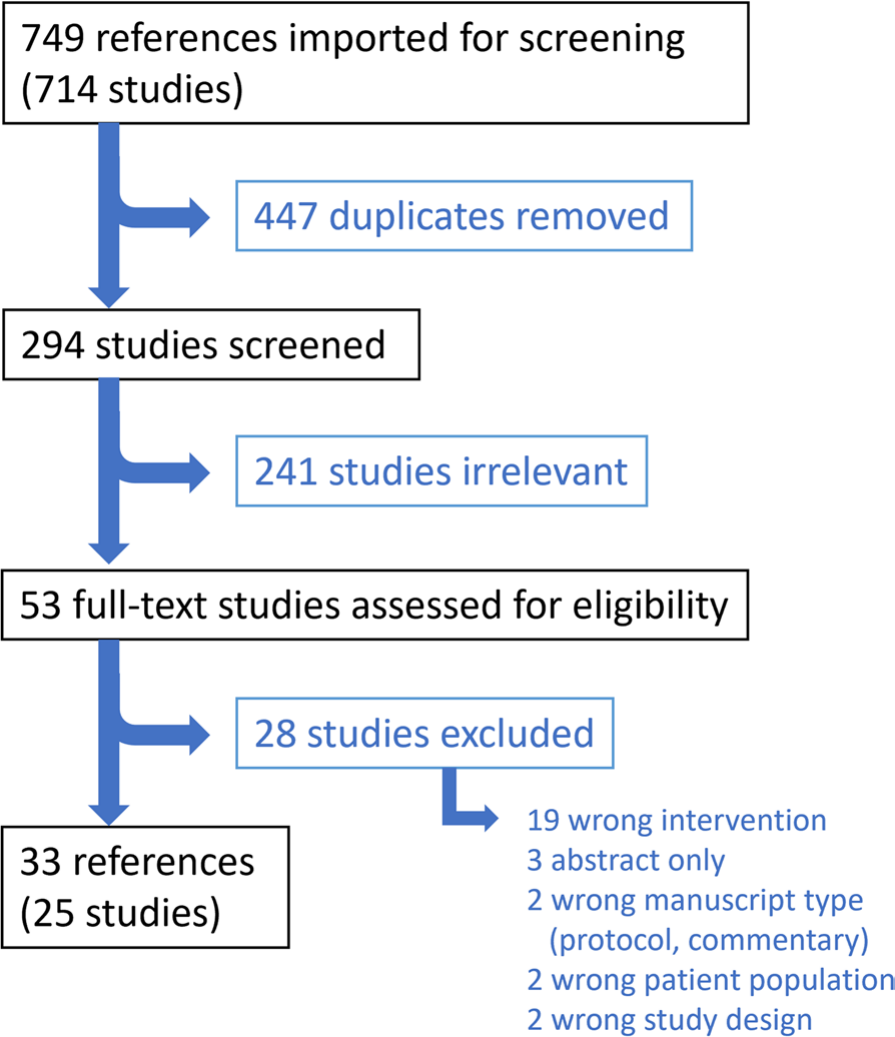
PRISMA diagram

Thirty-three articles from 25 studies met criteria for inclusion (see Fig. 1). Most studies were conducted in the United States (*n* = 20). Twenty studies focused on patient populations with heterogenous chronic pain. The remaining five studies focused on patients with chronic non-cancer pain, chronic neuromuscular disorders, chronic pelvic pain, chronic back pain, and rheumatoid arthritis (see Table 1).

**Table 1:** Manuscript details

No	Parent study	Author/year	Country	Setting	Condition	Sample	Study type	MMAT score
**1**	Chao et al. 2015 [38]	Chao et al. 2015 [38]	USA	Hospital	Non-malignant musculoskeletal pain	50 patients	Mixed methods	100
**2**	Clare et al. 2019 [39]	Clare et al. 2019 [39]	UK	Community Health Center (primary care)	Chronic pelvic pain	26 patients	Quantitative (Non-randomized)	60
**3**	Cornelio-Flores et al. 2018 [40]	Cornelio-Flores et al. 2018 [40]	USA	FQHC; Hospital	Non-specified chronic pain	19 patients	Mixed methods	80
**4**	Donovan et al. 1999 [41]	Donovan et al. 1999 [41]	USA	HMO	Non-specified chronic pain	237 patients	Mixed methods	0
**5**	Gardiner et al. 2014 [42]	Dresner et al. 2016 [43]	USA	Hospital	Non-specified chronic pain; Depression	19 patients	Qualitative	100
		Gardiner et al. 2014 [42]	USA	Hospital	Non-specified chronic pain	65 patients	Mixed methods	80
		Lestoquoy et al. 2017 [44]	USA	FQHC; Hospital	Non-specified chronic pain; Depression	20 patients	Qualitative	100
**6**	Gardiner, Luo, et al. 2019 [45]	Gardiner, Lestoquoy, et al. 2019 [46]	USA	FQHC; Hospital	Non-specified chronic pain	205 patients	Quantitative (Randomized Controlled Trial)	80
		Gardiner, Luo, et al. 2019 [45]	USA	FQHC; Hospital	Non-specified chronic pain		Quantitative (Randomized Controlled Trial)	80
		Nephew et al. 2022 [47]	USA	FQHC	Non-specified chronic pain; Depression	159 patients	Quantitative (Randomized Controlled Trial)	80
**7**	Geller et al. 2015 [48]	Geller et al. 2015 [48]	USA	FQHC	Non-specified chronic pain	42 patients	Quantitative (Non-randomized)	40
**8**	Harpole et al. 2003 [49]	Harpole et al. 2003 [49]	USA	HMO	Chronic headache	54 patients	Quantitative (Non-randomized)	40
**9**	Haun et al. 2020 [50]	Haun et al. 2020 [50]	USA	VA Medical Center	Non-specified chronic pain	201 patients	Quantitative (Non-randomized)	60
**10**	Mehl-Madrona et al. 2016 [51]	Mehl-Madrona et al. 2016 [51]	USA	Community Health Center (primary care)	Non-specified chronic pain	42 patients	Quantitative (Non-randomized)	60
**11**	Meriwether, Vellenga, Panter, et al. 2022 [52]	Meriwether, Vellenga, Panter, et al. 2022 [52]	USA	Allopathic Outpatient (specialty care)	Interstitial Cystitis/Bladder Pain syndrome (ICBPS)	45 patients	Qualitative	100
		Meriwether, Vellenga, Ravichandran, et al. 2022 [53]	USA	Allopathic Outpatient (specialty care)	Interstitial Cystitis/Bladder Pain syndrome (ICBPS)	45 patients	Quantitative (Non-randomized)	60
**12**	Miller et al. 2004 [54]	Miller et al. 2004 [54]	USA	Community Health Center (primary care)	Chronic disease diagnosis	28 patients	Mixed methods	100
**13**	Moitra et al. 2011 [55]	Moitra et al. 2011 [55]	USA	Community Health Center (primary care)	Non-specified chronic pain	50 patients	Mixed methods	0
**14**	Rayburn et al. 2017 [56]	Rayburn et al. 2017 [56]	USA	Hospital	Chronic back pain	98 patients	Quantitative (Descriptive)	0
**15**	Romanelli et al. 2017 [57]	Romanelli et al. 2017 [57]	USA	Community Health Center (primary care)	Non-specified chronic pain	130 patients	Quantitative (Non-randomized)	100
**16**	Roth et al. 2021 [58]	Roth et al. 2021 [58]	USA	Allopathic Outpatient (specialty care)	Non-specified chronic pain	15 patients	Qualitative	100
**17**	Seesing et al. 2014 [59]	Seesing et al. 2014 [59]	Netherlands	Allopathic Outpatient (specialty care)	Chronic neuromuscular disorders	272 patients	Quantitative (Randomized Controlled Trial)	80
		Seesing et al. 2015 [60]	Netherlands	Allopathic Outpatient (specialty care)	Chronic neuromuscular disorders	272 patients	Quantitative (Randomized Controlled Trial)	60
**18**	Shojania et al. 2010 [61]	Shojania et al. 2010 [61]	Canada	Allopathic Outpatient (specialty care)	Rheumatoid arthritis	19 patients	Mixed methods	60
**19**	Smith et al. 2016 [62]	Smith et al. 2016 [62]	Australia	Hospital	Chronic non-cancer pain	211 patients	Quantitative (Randomized Controlled Trial)	60
**20**	Spelman et al. 2017 [63]	Spelman et al. 2017 [63]	USA	VA Medical Center	Non-specified chronic pain	24 patients	Quantitative (Non-randomized)	0
**21**	Taube et al. 2021 [64]	Taube et al. 2021 [64]	USA	VA Medical Center	Non-specified chronic pain	16 patients	Quantitative (Non-randomized)	20
**22**	Thompson-Lastad et al. 2019 [65]	Thompson-Lastad et al. 2018 [66]	USA	FQHC; Hospital; VA Medical Center	Non-specified chronic pain	25 patients 28 clinicians/staff	Qualitative	100
		Thompson-Lastad et al. 2019 [65]	USA	FQHC; Hospital; VA Medical Center	Non-specified chronic pain	57 clinicians/staff	Quantitative (Descriptive)	60
		Thompson-Lastad et al. 2020 [67]	USA	FQHC; Hospital; VA Medical Center	Non-specified chronic pain	46 clinicians/staff	Qualitative	100
**23**	Wile et al. 2021 [68]	Wile et al. 2021 [68]	USA	Community Health Center (primary care)	Chronic non-cancer pain	19 patients	Qualitative	100
**24**	Wong et al. 2015 [69]	Wong et al. 2015 [69]	Canada	Community Health Center (primary care)	Non-specified chronic pain	29 patients 34 clinicians/staff	Qualitative	100
**25**	Znidarsic et al. 2021 [70]	Znidarsic et al. 2021 [70]	USA	Integrative Medicine Clinic	Chronic non-cancer pain	178 patients	Quantitative (Descriptive)	80

### Quality assessment

Eight manuscripts were qualitative, six were quantitative randomized controlled trials, nine were quantitative non-randomized, three were quantitative descriptive, and seven were mixed methods (see Tables 2, 3, 4, 5 and 6). Quality assessment ratings for articles ranged from 0 to 100%. The four studies that received ratings of 0 failed to do one or more of the following: present clear research questions, collect data that allowed them to answer their research questions, or provide adequate rationale for using a mixed methods design. On average, manuscripts with qualitative methods held the highest ratings, indicating high quality and low risk of bias, and manuscripts with quantitative, non-randomized methods held the lowest. The largest contributors to lower MMAT ratings included incomplete outcome data, failure to account for confounding variables in study design and analysis, nonresponse bias, and participants not being representative of the target population.

**Table 2:** MMAT bias assessment. Bias assessment for qualitative studies (*n* = 8)

	**Screening questions**		**Qualitative bias assessment questions**					**Rating (%)**
	Are there clear research questions?	Do the collected data allow to address the research questions?	Is the qualitative approach appropriate to answer the research question?	Are the qualitative data collection methods adequate to address the research question?	Are the findings adequately derived from the data?	Is the interpretation of results sufficiently substantiated by data?	Is there coherence between qualitative data sources, collection, analysis and interpretation?	
Dresner et al. 2016 [43, 71]	Yes	Yes	Yes	Yes	Yes	Yes	Yes	100
Meriwether, Vellenga, Panter, et al. 2022 [52]	Yes	Yes	Yes	Yes	Yes	Yes	Yes	100
Roth et al. 2021 [58]	Yes	Yes	Yes	Yes	Yes	Yes	Yes	100
Wile et al. 2021 [68]	Yes	Yes	Yes	Yes	Yes	Yes	Yes	100
Wong et al. 2015 [69]	Yes	Yes	Yes	Yes	Yes	Yes	Yes	100
Lestoquoy et al. 2017 [44]	Yes	Yes	Yes	Yes	Yes	Yes	Yes	100
Thompson-Lastad et al. 2018 [66]	Yes	Yes	Yes	Yes	Yes	Yes	Yes	100
Thompson-Lastad et al. 2020 [67]	Yes	Yes	Yes	Yes	Yes	Yes	Yes	100

**Table 3:** MMAT bias assessment. Bias assessment for quantitative (randomized controlled trial) studies (*n* = 6)

	**Screening questions**		**Quantitative (randomized controlled trial) bias assessment questions**					**Rating (%)**
	Are there clear research questions?	Do the collected data allow to address the research questions?	Is randomization appropriately performed?	Are the groups comparable at baseline?	Are there complete outcome data?	Are outcome assessors blinded to the intervention provided?	Did the participants adhere to the assigned intervention?	
Gardiner, Luo, et al. 2019 [45]	Yes	Yes	Yes	Yes	Yes	Yes	No	80
Gardiner, Lestoquoy, et al. 2019 [46]	Yes	Yes	Can’t Tell	Yes	Yes	Yes	Yes	80
Nephew et al. 2022 [47]	Yes	Yes	Yes	Yes	Yes	Not Assessable	Yes	80
Smith et al. 2016 [62]	Yes	Yes	Yes	No	No	Yes	Yes	60
Seesing et al. 2014 [59]	Yes	Yes	Yes	Yes	Yes	Not Assessable	Yes	80
Seesing et al. 2015 [60]	Yes	Yes	Yes	Yes	Yes	No	Not Assessable	60

**Table 4:** MMAT bias assessment. Bias assessment for quantitative (non-randomized) studies (*n* = 9)

	**Screening questions**		**Quantitative (non-randomized) bias assessment questions**					**Rating (%)**
	Are there clear research questions?	Do the collected data allow to address the research questions?	Are the participants representative of the target population?	Are measurements appropriate regarding both the outcome and intervention (or exposure)?	Are there complete outcome data?	Are the confounders accounted for in the design and analysis?	During the study period, is the intervention administered (or exposure occurred) as intended?	
Clare et al. 2019 [39]	Yes	Yes	Yes	Yes	No	No	Yes	60
Geller et al. 2015 [48]	Yes	Yes	Not Assessable	Yes	No	No	Yes	40
Harpole et al. 2003 [49]	Yes	Yes	Not Assessable	Yes	No	Not Assessable	Yes	40
Haun et al. 2020 [50]	Yes	Yes	No	Yes	No	Yes	Yes	60
Mehl-Madrona et al. 2016 [51]	Yes	Yes	Yes	Yes	No	Not Assessable	Yes	60
Meriwether, Vellenga, Ravichandran, et al. 2022 [53]	Yes	Yes	Yes	Yes	No	Yes	No	60
Taube et al. 2021 [64]	Yes	Yes	Not Assessable	Not Assessable	Yes	No	No	20
Spelman et al. 2017 [63]	No	No	N/A	N/A	N/A	N/A	N/A	0
Romanelli et al. 2017 [57]	Yes	Yes	Yes	Yes	Yes	Yes	Yes	100

**Table 5:** MMAT bias assessment. Bias assessment for quantitative (descriptive) studies (*n* = 3)

	**Screening questions**		**Quantitative (descriptive) bias assessment questions**					**Rating (%)**
	Are there clear research questions?	Do the collected data allow to address the research questions?	Is the sampling strategy relevant to address the research question?	Is the sample representative of the target population?	Are the measurements appropriate?	Is the risk of nonresponse bias low?	Is the statistical analysis appropriate to answer the research question?	
Znidarsic et al. 2021 [70]	Yes	Yes	Yes	Yes	Yes	No	Yes	80
Rayburn et al. 2017 [56]	No	No	N/A	N/A	N/A	N/A	N/A	0
Thompson-Lastad et al. 2019 [65]	Yes	Yes	Yes	Not Assessable	Yes	Not Assessable	Yes	60

**Table 6:** MMAT bias assessment. Bias assessment for mixed methods studies (*n* = 7)

	**Screening questions**		**Mixed methods bias assessment questions**					**Rating (%)**
	Are there clear research questions?	Do the collected data allow to address the research questions?	Is there an adequate rationale for using a mixed methods design to address the research question?	Are the different components of the study effectively integrated to answer the research question?	Are the outputs of the integration of qualitative and quantitative components adequately interpreted?	Are divergences and inconsistencies between quantitative and qualitative results adequately addressed?	Do the different components of the study adhere to the quality criteria of each tradition of the methods involved?	
Chao et al. 2015 [38]	Yes	Yes	Yes	Yes	Yes	Yes	Yes	100
Cornelio-Flores et al. 2018 [40]	Yes	Yes	Yes	Yes	Yes	Yes	No	80
Donovan et al. 1999 [41]	Yes	Yes	N/A	N/A	N/A	N/A	N/A	0
Gardiner et al. 2014 [42]	Yes	Yes	Yes	Yes	Yes	Yes	No	80
Moitra et al. 2011 [55]	Yes	Yes	Not Assessable	Not Assessable	No	Not Assessable	No	0
Miller et al. 2004 [54]	Yes	Yes	Yes	Yes	Yes	Yes	Yes	100
Shojania et al. 2010 [61]	Yes	Yes	Yes	Yes	Yes	Not Assessable	No	60

### Description of participants involved

 Articles included data from patients (*n* = 23), clinicians/staff (*n* = 2), or both patients and clinicians/staff (*n* = 2) (see Table 7). The mean age of participants ranged from 40 to 62 years old. In most studies, females comprised the large majority of participants (75% on average). Twenty articles included the racial or ethnic makeup of their participants. The participants in these articles, on average, were 41% non-Hispanic white, 24% Black or African American, 30% Hispanic, 1% Asian American or Pacific Islander, 5% Native American, and 9% other or unknown. Several studies explicitly focused on reaching racially and ethnically under-represented patient populations [40, 46, 48, 71].

**Table 7:** Participant demographics

Author	**Age**		**Race/ethnicity (%)**						**Gender (%)**		**Education**			
	Mean	Range	Black or African American	Hispanic	White	Asian American or Pacific Islander	Native American	Other or Unknown	Male	Female	Less than high school	High school	Some college	College graduate
Clare et al. 2019 [39]	45	N/A	N/A	N/A	N/A	N/A	N/A	N/A	8	92	N/A	N/A	N/A	N/A
Chao et al. 2015 [38]	40	23–63	32	28	24	8	0	8	0	100	0	12	38	50
Cornelio-Flores et al. 2018 [40]	52	N/A	0	100	0	0	0	0	11	89	35	24	29	12
Donovan et al. 1999 [41]	N/A	N/A	N/A	N/A	N/A	N/A	N/A	N/A	N/A	N/A	N/A	N/A	N/A	N/A
Dresner et al. 2016 [43, 71]	53	N/A	63	11	26	0	0	0	N/A	N/A	N/A	N/A	N/A	N/A
Gardiner et al. 2014 [42]	51	N/A	60	9	23	0	0	0	32	68	20	19	47	14
Gardiner, Lestoquoy, et al. 2019 [46]	50	22–84	60	15	16	0.3	0	24	17	83	N/A	N/A	N/A	N/A
Gardiner, Luo, et al. 2019 [45]	50	22–84	56	14	19	0	0	36	14	86	18	34	34	14
Geller et al. 2015 [48]	51	N/A	0	100	0	0	0	0	0	100	N/A	N/A	N/A	N/A
Harpole et al. 2003 [49]	40	N/A	0	93	0	0	0	7	20	80	N/A	N/A	N/A	N/A
Haun et al. 2020 [50]	52	N/A	40	13	56	3	1	0	0	100	N/A	N/A	N/A	N/A
Lestoquoy et al. 2017 [44]	47	26–60	50	15	25	0	5	20	25	75	N/A	N/A	N/A	N/A
Mehl-Madrona et al. 2016 [51]	46	N/A	N/A	N/A	N/A	N/A	N/A	N/A	40	60	0	0	0	41.5
Moitra et al. 2011 [55]	45	N/A	2	4	94	0	0	0	42	58	26	28	36	10
Meriwether, Vellenga, Panter, et al. 2022 [52]	52	35–86	0	43	57	0	43	0	0	100	0	54	0	46
Meriwether, Vellenga, Ravichandran, et al. 2022 [53]	52	35–86	0	43	57	0	43	0	0	100	0	54	0	46
Miller et al. 2004 [54]	50	40–64	14	71	7	7	0	0	0	100	N/A	N/A	N/A	N/A
Nephew et al. 2022 [47]	50	24–84	58	14	16	0	0	26	17	83	19	30	31	10
Rayburn et al. 2017 [56]	N/A	N/A	N/A	N/A	N/A	N/A	N/A	N/A	N/A	N/A	N/A	N/A	N/A	N/A
Romanelli et al. 2017 [57]	N/A	18–65	N/A	N/A	N/A	N/A	N/A	N/A	44	56	N/A	N/A	N/A	N/A
Roth et al. 2021 [58]	N/A	N/A	N/A	N/A	N/A	N/A	N/A	N/A	N/A	N/A	N/A	N/A	N/A	N/A
Seesing et al. 2014 [59]	51	N/A	N/A	N/A	N/A	N/A	N/A	N/A	53	47	N/A	N/A	N/A	46
Seesing et al. 2015 [60]	51	N/A	N/A	N/A	N/A	N/A	N/A	N/A	53	47	N/A	N/A	N/A	46
Shojania et al. 2010 [61]	52	45–76	N/A	N/A	N/A	N/A	N/A	N/A	32	68	N/A	N/A	N/A	N/A
Smith et al. 2016 [62]	49	20–72	N/A	N/A	N/A	N/A	N/A	N/A	32	68	N/A	N/A	N/A	N/A
Spelman et al. 2017 [63]	N/A	N/A	N/A	N/A	N/A	N/A	N/A	N/A	N/A	N/A	N/A	N/A	N/A	N/A
Taube et al. 2021 [64]	59	N/A	0	0	100	0	0	0	100	0	N/A	N/A	N/A	N/A
Thompson-Lastad et al. 2018 [66]	58	N/A	N/A	N/A	N/A	N/A	N/A	N/A	28	72	8	28	36	25
Thompson-Lastad et al. 2019 [65]	50	N/A	N/A	11	83	7		9	10	90	N/A	N/A	N/A	N/A
Thompson-Lastad et al. 2020 [67]	N/A	N/A	N/A	N/A	N/A	N/A	N/A	N/A	24	76	N/A	N/A	N/A	N/A
Wile et al. 2021 [68]	54	36–65	0	11	89	0	0	5	53	47	N/A	N/A	N/A	N/A
Wong et al. 2015 [69]	62	N/A	0	0	55	0	0	45	36	66	N/A	N/A	N/A	N/A
Znidarsic et al. 2021 [70]	62	53–69	25	0	69	1	1	3	13	87	2	8	23	67
**Average**	**51**	**22–86**	**24**	**30**	**41**	**1**	**5**	**9**	**25**	**75**	**12**	**26**	**25**	**33**

### Characteristics of GMV innovations

Most studies evaluated group visit models with a pre-specified number of sessions (as opposed to some group visit models which conduct meetings indefinitely) (See Table 8). Studies reported on group visits delivered in English, Spanish, Dutch, Chinese, and Korean. Physicians facilitated the GMV in 21 studies, often in collaboration with physical therapists, nurses, physician assistants/nurse practitioners, and complementary and integrative practitioners. Eleven studies mentioned that group visits were billed fee-for-service via ICD-10 codes. Six studies describe specific health insurance coverage.

**Table 8:** Characteristics of group medical visit design and delivery

Author	Group design	Language	Intervention leaders	Confidentiality	Billing	Insurance
Clare et al. 2019 [39]	10 group visits; 3 h/session; 1 session/week; 2 follow-up visits	English	Mental health therapist; Nurse; Physiotherapist	Not documented	Yes	National Health Service
Chao et al. 2015 [38]	10 group visits; 2 h/session; 1 session/month	English	Nurse practitioner; Physician assistant	Not documented	Not documented	Not documented
Cornelio-Flores et al. 2018 [40]	9 group visits; 2 h/session; 1 session/week;	Spanish	Physician; CIH provider	Yes (verbal)	Not documented	Not documented
Donovan et al. 1999 [41]	6 group visits; 1–2 h/session	English	Physician; Physician assistant/Nurse practitioner; Social worker; Pharmacist	No	Not documented	Not documented
Dresner et al. 2016 [43, 71]	8 group visits; 1 session/week; 2 h/session	English	Physician; Research coordinator	Yes (written)	Not documented	Not documented
Gardiner et al. 2014 [42]	8 group visits; 1 session/week; 2 h/session	English	Physician; Mindfulness instructor; CIH providers	Yes (verbal and written)	Yes	Not documented
Gardiner, Lestoquoy, et al. 2019 [46]	8 group visits; 1 session/week; 2 h/session	English	Physician; Physician assistant/nurse practitioner; Nurse; physical therapist; Mental health therapist	Yes (verbal and written)	Yes	Not documented
Gardiner, Luo, et al. 2019 [45]	10 group visits; 1 session/week; 2.5 h/session	English	Physician; Minfulness instructor; Yoga teacher	Yes (verbal and written)	Not documented	Not documented
Geller et al. 2015 [48]	Open enrollment group visits; 1 session/week; 1.5 h/session	Not documented	Physician; CIH provider	Not documented	Not documented	Not documented
Harpole et al. 2003 [49]	1 group visit; 2 h/session	English	Physician; Physician assistant/Nurse practitioner	Not documented	Not documented	Not documented
Haun et al. 2020 [50]	14 group visits; 1 session/week; 2 h/session	English	Physician; Physician assistant/nurse practitioner; Physical therapist; Nutritionist; Yoga teacher	Not documented	Not documented	Veterans Affairs (Tricare)
Lestoquoy et al. 2017 [44]	8 group visits; 1 session/week; 2 h/session	Not documented	Physician; Mindfulness instructor	Not documented	Not documented	Not documented
Mehl-Madrona et al. 2016 [51]	12 group visits; 2 sessions/month; 2 h/session	English	Physician; Nurse; Mental health therapist	Not documented	Yes (billed as group therapy)	Not documented
Moitra et al. 2011 [55]	1 group visit; 1.25 h/session	English	Physician; Mental health therapist	Not documented	Yes (billed for complexity of care)	Not documented
Meriwether, Vellenga, Panter, et al. 2022 [52]	12 group visits; 1 session/month; 2–3 h/session	English	Nurse; Midwife	Not documented	Yes (fee-for-service)	Not documented
Meriwether, Vellenga, Ravichandran, et al. 2022 [53]	12 group visits; 1 session/month; 2–3 h/session	English	Nurse; Midwife	Not documented	Yes (fee-for-service)	Not documented
Miller et al. 2004 [54]	6 group visits over 9 months; 1.5 h/session	Not documented	Physician; Physician assistant/Nurse practitioner	Yes (verbal)	Not documented	Not documented
Nephew et al. 2022 [47]	10 group visits; 1 session/week; 2.5 h/session	English	Physician; Yoga teacher; Mindfulness instructor	Not documented	Not documented	Not documented
Rayburn et al. 2017 [56]	5 group visits; 1 session/month	English; Spanish	Physician; Nurse; Medical assistant	Not documented	Yes (fee-for-service)	Not documented
Romanelli et al. 2017 [57]	1 group visit; 1.5 h/session	English	Physician; medical assistant	Not documented	Yes	Medicare/Medicaid; Private Insurance
Roth et al. 2021 [58]	8 group visits; 1 session/week; 2 h/session	English	Physician; Yoga teacher; Mindfulness instructor	Yes (verbal)	Not documented	Not documented
Seesing et al. 2014 [59]	1 group visit; 1.5–2 h/session	Dutch	Physician; Medical assistant	Not documented	Not documented	Not documented
Seesing et al. 2015 [60]	1 group visit; 1.5–2 h/session	Dutch	Physician; Nurse	Yes (written)	Not documented	Not documented
Shojania et al. 2010 [61]	6 group visits; 3.5 h/session	English	Physician; Nurse; Physical therapist; Occupational therapist; Nutritionist; Pain specialist	Yes (written)	Yes ($60 CAD per patient)	Universal Health Coverage
Smith et al. 2016 [62]	1 group visit; 5 h/session	English	Physician; Nurse; Physical therapist; Mental health therapist; Pain specialist	Not documented	Not documented	Not documented
Spelman et al. 2017 [63]	1 group visit; 1.5 h/session	Not documented	Physician	Not documented	Not documented	Not documented
Taube et al. 2021 [64]	6 group visits; 1 session/month; 1.5 h/session	English	Physician; Nurse; Physical therapist; Nutritionist; Medical assistant; Clinical pharmacist; Occupational therapist	Not documented	Not documented	Not documented
Thompson-Lastad et al. 2018 [66]	20 group visits; 8 clinical sites; 4 organizations	English; Spanish	Physician; Physician assistant/Nurse practitioner; Medical assistant; Health educator	Not documented	Yes (ICD-10 codes)	Not documented
Thompson-Lastad et al. 2019 [65]	Variable	Spanish; Chinese; Korean	Physician; Physician assistant/Nurse practitioner; Physical therapist; Medical assistant; Acupuncturist; Yoga teacher; CIH providers	Not documented	Yes (ICD-10 codes)	Veteran’s benefits; Free or discounted care
Thompson-Lastad et al. 2020 [67]	22 group visit programs; 11 clinical sites; 6 organizations	English; Spanish	Physician; Physician assistant/Nurse practitioner; Physical therapist; Medical assistant; Acupuncturist; CIH providers	Not documented	Yes (ICD-10 codes)	Not documented
Wile et al. 2021 [68]	Variable	English	Variable	Yes (verbal)	No	Not documented
Wong et al. 2015 [69]	Weekly to quarterly group visit programs; 1–1.5 h/session	English	Physician; Physician assistant/Nurse practitioner; Nurse; Medical assistant; Community Health Worker	Not documented	Not documented	British Columbia’s Alternative Payment Plan
Znidarsic et al. 2021 [70]	8 group visits; 1 session/week; 3 h/session	English	Physician; Mental health therapist; CIH providers	Not documented	Not documented	Not documented

### Determinants of GMV innovations

**Fig. 2:**
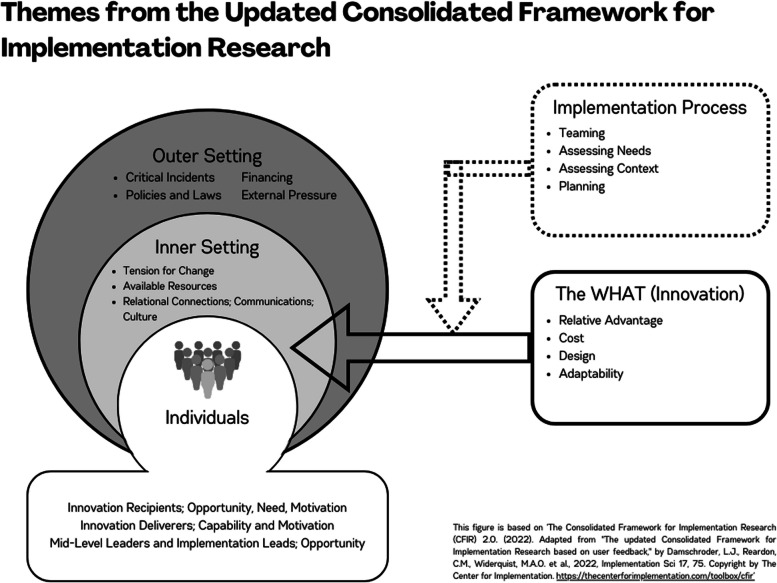
Themes from the updated consolidated framework for implementation research

Below, we present findings on determinants of GMV implementation, within each of the five CFIR domains (see Fig. 2). Only qualitative data on determinants of implementation was extracted, no included studies measured barriers and facilitators to implementation quantitatively. A summary of themes and selected illustrative quotes and data sources within the original manuscripts is presented in Table 9. A full accounting of source quotes and data sources within the original manuscripts is presented in Appendix B.

**Table 9:** CFIR determinants by domain

Determinants	Quotes	Data source
**Innovation**		
Relative advantage	“perception of having more time with your doctor and the collaborative relationship among participants to learn new skills or knowledge about different health topics.”	Cornelio-Flores, 2018 [40] pg. 130 Results, focus group data with patients
	“The GMV structure also helped to neutralize the inherent power imbalance between patient and provider. GMVs were more interactive, allowing patients to gain information from their providers but also to listen and share their day-to-day management strategies with each other”	Wong, 2015 [69] pg. 36 Results, qualitative interviews with clinicians and patients
Cost	“We continue to have these programs today and run them in a financially self-sustaining way. There is little extra financing, so group programs are limited to twice a week.”	Geller, 2015 [48] pg. 31, Discussion
	“GMVs averaged 12 people per session over 2 h[hours]…Thus, improved care at a minimum broke even financially”	Mehl-Madrona, 2016 [51] pg. 624, Results, cost data
Adaptability	“Participants mentioned they thought having diversity within the Centering group was beneficial, particularly regarding age. Diversity, in their view, engendered a sense of comfort, lack of judgment, and a belief that ICBPS could affect anybody of any background”	Meriwether, 2022 [52] p.696, Results, qualitative focus group data with patients
	“it may be more challenging to provide group care in a mixed ethnicity setting and that additional effort is needed to develop interventions that provide group care across cultural boundaries”	Miller, 2004 [54] p.223, Discussion
**Inner setting**		
Tension for change: alternative treatment options	“The development of clinic policies for opioid prescribing and increased consistency across prescribers were described as increasing cohesiveness among staff”	Moitra, 2011 [55] pg. 157, Results, qualitative assessment with staff
	“Through our analysis of IMGVs for chronic pain management, we found that uncertainties surrounding the treatment of pain in the midst of the opioid crisis created similar space for the deployment of emerging forms of clinical practice, including IMGVs for chronic pain”	Thompson-Lastad, 2020 [67] pg. 2, Introduction
Communications: referral systems	“increasing eligible patient attendance in the intervention group was challenging, requiring modifications that targeted both clinicians and patients such as list distribution and pro-active e-mail reminders to clinicians, as well as a letter to patients informing them about the group.”	Spelman, 2017 [63] p. 2328, Discussion
	“respondents emphasized the need for adequate staffing and institutional support for patient recruitment, such as staff to make reminder phone calls to patients and to open facilities during evening hours when more patients are available.” ( pg. 5)	Thompson-Lastad, 2019 [65] Results, survey of clinicians and staff
Available resources: previous culture supporting groups	“Stakeholders lack of familiarity with IMGV”	Roth 2021 [58] p.S-75, Results, qualitative interviews with clinicians, administrators, and staff
	“designing and implementing the group visit within our practice setting required relatively little time and effort… This low investment was likely due to the fact that other group visits for conditions such as diabetes and opioid/chronic pain education have been implemented at our site.”	Spelman, 2017 [63] p.2328, Discussion
**Outer Setting**		
Financing	“Our institution has successfully billed for the program, reimbursed in the same way as a regular clinic visit”	Rayburn 2017 pg. 12, Discussion
	“The absence of frequent, long-term CIH [complementary and integrative health] treatment despite clinician and patient interest was a direct result of the fact that Medicaid and Medicare did not generally reimburse for CIH, even approaches that were recommended by major medical organizations”	Thompson-Lastad, 2020 [67] p. 258, Results, qualitative interviews with clinicians and staff
Critical incidents; external pressure: opioid crisis and COVID-19 pandemic	“26 [patients] left because they failed to maintain their pain contract and were being tapered off opiates and found other care”	Mehl-Madrona, 2016 [51] pg. 623, Results, administrative data
	“Because interviews were conducted during the COVID-19 pandemic, implementation strategies were designed to be conducted either in person or virtually (with some activities specified as telehealth-only).”	Roth 2021 [58], p.S-77, Results, qualitative interviews with clinicians and staff
Policies and laws: licensing/credentialing of CIH practitioners	“Providers also had specific questions about staffing IGMVs with appropriately trained clinicians and support staff, and implementing and billing for complementary health approaches”	Thompson-Lastad 2019 [65] p. 6, Results, survey of clinicians and staff
	“Clinical facilitator buy-in to deliver IMGV Cofacilitator availability to conduct IMGV during clinical hours Nursing staff not trained in check-in, Clinical facilitator’s knowledge of IMGV eligibility, Cofacilitator’s preparation to lead IMGVs”	Roth, 2021 [58] p. S-75, Results, qualitative interviews with clinicians, administrators, and staff
**Individuals**		
Innovation recipients: opportunity	“The main reasons for declining was scheduling concerns (e.g. work during the day, too much of a time commitment, *n* = 66), personal preferences about groups/ social contact (not wanting to be part of a group or not liking interacting with others, *n* = 33), Other reasons for declining included: medical concerns (surgeries scheduled, trouble with mobility, too many appointments/other medical commitments, *n* = 10),…childcare related problems (*n* = 8), transportation barriers (*n* = 10), and not speaking English as a first language (*n* = 2)”	Gardiner 2019 [45], Explore, p.219, Results, interview data with patients
	“Participants expressed that most barriers to joining Centering were logistical, such as not being able to make the meeting time, use Zoom, or attend in person… although the convenience of doing Centering over Zoom was a positive aspect, meeting in person would have added more depth to their interactions and allowed them to connect socially with their peers”	Meriwether, 2022, Results, qualitative focus group data with patients
Innovation recipients; need	“Qualitative data suggest that increased self-efficacy and improvement in symptoms (eg, mood, sleep disorders) may help mediate improvement in pain level”	Gardiner, 2014 [42] pg. 24, Results, qualitative interviews with patients
	“Providers noted how IGMVs allowed patients to share expertise and support one another, which several described as patient-empowering. Providers’ favorite aspects of IGMVs included positive changes in patient–provider relationships. They also noted improvements in patients’ physical and mental health, which they attributed to both complementary health approaches and peer support”	Thompson-Lastad 2019 [65], p. 5, Results, survey of clinicians and staff
Implementation deliverers: community/context expertise	“It is the skills of group facilitation and management that become more important than the ultimate curriculum.”	Geller, 2015 [48] p. 31, Discussion
	“the group changed when physicians participate: They were our equals, they weren’t physicians, you know…They taught. …They came down on our level and…When we do the exercise, they do the exercise.”	Lestoquoy, 2017 [44] p. 37, Results, focus group data with patients
Innovation recipients: motivation	“They thought people should only pay for medical advice from a health care provider and not support from peers.” “Billing for the Centering visits was something that not only upset patients but might be a barrier to entry or diversity of the group.”	Meriwether, 2022, results, qualitative focus group data from participants
	“At these group visits, patients were engaged, valued the experience, and all requested prescriptions for the naloxone kit, suggesting the small out-of-pocket cost for veterans was not a barrier.”	Spelman, 2017 [63] p.2328, Discussion
**Process**		
Assessing needs	“Stratification was most visible in limited access to IGMVs for non-English speakers and people with severe mental health conditions, with notable exceptions… Individual clinicians’ comfort with mental health conditions shaped which patients were welcomed into IGMVs”	Thompson-Lastad 2020 [67] p. 258, Results, qualitative interview data with clinicians and staff
	“Providers also identified patients whom they felt were less suited to participating in GMVs, including those who were hard of hearing, had limited English-speaking skills or cognitive deficits or were uncomfortable in groups”	Wong 2015 [69] p. 37, Results, qualitative interview data with clinicians
Teaming, assessing context, planning	“Involve support staff, including administrative and billing staff, in planning because some ideas may not be feasible under managed care or with certain insurance payors.”	Moitra 2011 [55] p. 158, Discussion

#### Innovation characteristics

##### **Relative advantage**

Thirteen studies described the relative advantage of providing GMVs compared to other forms of chronic pain care [40, 44, 51, 53, 56, 57, 60, 61, 63, 64, 66, 68, 69]. Eight studies present survey data, interview data, or ethnographic observations in the “Results” section and five studies discussed relative advantages in the “Discussion” section. Patients (innovation recipients) and providers (innovation deliverers) described how their experience receiving or delivering care was improved in GMVs. Groups allowed providers to spend more time with their patients (typically GMVs are one to three hours long), provided access to complementary and integrative therapies, and improved patient-provider relationships.

One article also discussed the benefits of GMVs “over other types of group encounters,” including having groups facilitated by a billing provider who can document the visit in the electronic health record, and provide care coordination with other providers [57]. Another study described how the innovation “outperformed provider education,” suggesting its relative advantage over another commonly used innovation [63].

##### Cost to innovation recipients and health system

Four studies specifically discuss the cost of the innovation; either to the organization where group medical visits are implemented, or the patients who are receiving care [48, 51, 60, 62]. Two manuscripts mention that the GMV programs are either “financially self-sustaining [48]” or “broke even financially [51].” Two articles provided supporting cost data [51, 60]. Two articles discussed contextual information regarding cost in the “Discussion” section [48, 62]. Only one article included a cost-effectiveness analysis, which concluded that the group medical visit program “was more cost-effective than an individual appointment when a group size of more than 6 patients was maintained [60].” Another article addressed the issue of overbooking groups to minimize the financial impact of patients not attending [62].

Several studies reported on measures related to cost, including emergency room utilization [45, 49, 64], healthcare utilization [39, 54, 55], and wait times for appointments [62], all noting that GMVs reduced high-cost healthcare use and improved access to appointments.

##### Design and adaptability: population served

Two manuscripts described how the relative homogeneity or heterogeneity of the patients impacted the success of the innovation [52, 54]. The manuscripts come to differing conclusions about whether having an ethnically diverse population of innovation recipients is beneficial or not for the success of the innovation, with patients reporting that diversity was a benefit in one study [52], and the authors conjecturing that a mixed ethnicity setting may be challenging in the other study [54].

#### Inner setting

##### Tension for change: opportunities for treatment options

Two manuscripts reference the pressure of the opioid crisis leading to changes in clinic policy that created an opportunity for changing clinical treatment of chronic pain [55, 67]. Internal clinic policies created consistency and increased cohesiveness among staff [55], or may have provided an opportunity to offer new forms of clinical care [67].

##### Relational connections, communications, and culture: group recruitment and referral systems

Seven studies reported on the importance of referral networks and recruitment strategies within the clinical environment, though only two presented data to support this assertion [46, 48, 54, 55, 58, 63, 65]. Staffing to make reminder calls, physicians giving personal referrals to the group visit program, email reminders, and distributing lists of eligible patients to providers were all mentioned as important strategies for supporting innovation recipient recruitment.

##### Available resources: previous culture supporting groups

Seven studies discussed the importance of previous GMV programs in the organization [40, 42, 45, 48, 58, 63, 66]. Four manuscripts discussed GMVs for chronic pain that developed out of the same research group at Boston Medical Center and discussed how existing GMV programs helped to pave the way for additional research and iterations of GMVs for patients with chronic pain [40, 42, 43, 45]. An additional three manuscripts discussed how the presence of GMVs for other conditions at the clinical site, including obesity, diabetes, and group prenatal care, helped to lower the initial investment and reduce barriers to implementing chronic pain groups [48, 63, 66]. One manuscript presented data supporting showing how the lack of a previous culture supporting GMVs, and the lack of familiarity of the clinic’s providers and staff with the GMV model was a barrier to be overcome in successfully implementing groups [58].

#### Outer setting

##### Financing; policies and laws

Five studies reported on issues pertaining to financing and insurance reimbursement of GMVs, with three presenting qualitative data related to perceived financing challenges from the perspectives of clinicians and staff [55, 56, 65, 68, 69]. Group visit programs billed as group therapy or typical primary care visits, for instance, may not match the reimbursement providers receive for more complex pain consultation services [55]. One manuscript discussed the financial risk providers take by running group visit programs, as reimbursement is dependent on the number of patients enrolled [69]. One manuscript described successful billing practices [56], while another discussed insurance reimbursement as a barrier to GMVs and medical care in general [68].

Other financial barriers to group implementation concerned the burden participation in group visits placed on individual patients. One manuscript described how clinical staff expressed confusion over how to serve patients with high co-pays, the frequency of billing, and who was responsible for billing patients who participated in group programs [65].

One study referenced the challenges associated with compensating providers of nonpharmacological pain management services (e.g. acupuncturists), particularly because these services are not generally reimbursed by Medicare or Medicaid [65, 67].

##### Critical incidents and external pressure: opioid crisis and COVID-19 pandemic

Two studies mention challenges with implementation of GMVs pertaining to the ongoing opioid epidemic [51, 55]. Both studies describe challenges with enrolling or retaining patients in GMVs whose expectations around receiving opioid treatment did not match the policies of the clinic. Two studies briefly mentioned the COVID-19 pandemic, noting that both planning activities and GMVs could be conducted via telehealth due to the pandemic [58, 64].

##### Policies and laws: reimbursement of complementary and integrative health (CIH) practitioners

Two studies discuss challenges related to staffing GMVs with staff who are trained in complementary and integrative health (CIH) modalities [58, 65]. Thompson-Lastad [67] discussed how staff trained in a CIH modality such as acupuncture or yoga typically played multiple roles in their workplaces, primarily due to the lack of reimbursement to provide CIH services in individual visits.

#### Characteristics of individual

##### Innovation recipients: need

Ten studies reported on how group medical visits improved the innovation recipient’s quality of life, including improvements in self-efficacy, satisfaction, function, and improvements in physical and mental health [38, 42, 49, 50, 57, 61, 64, 65, 68, 70]. These improvements in well-being and personal fulfillment were attributed to the benefits of the innovation. One manuscript reported on innovation recipient’s negative expectations around group visits, including “fear the experience will be detrimental to their wellbeing due to possible contagion [62].”

##### Innovation recipients: opportunity

Ten manuscripts reported that patients had encountered logistical challenges in attending group medical visits. For in-person groups, barriers were largely related to transportation, scheduling, and health challenges making it difficult to attend. For telehealth groups, there were some challenges accessing technology and scheduling remained a challenge for some. Thus, innovation recipients (patients with chronic pain), lacked the opportunity or availability to fulfill their role in receiving the GMV.

Out of twenty five studies, eight mentioned that confidentiality was addressed within the group, six mentioned no concerns with confidentiality, seventeen did not document, and two manuscripts described intended innovation recipients who had concerns about privacy [61, 68], or feeling vulnerable or anxious in a group setting [52, 68].

##### Innovation recipients: motivation

Two studies discussed out-of-pocket costs from the patient perspective [52, 63]. When asked, patients were very opposed to paying a co-pay for a GMV, noting that they “perceived the group as a support group, noted that no other support groups cost money [52].” In another GMV, where innovation recipients were offered the overdose-reversing drug Nalaxone at a discount, the authors noted that the out-of-pocket cost was not a barrier for innovation recipients, and that they were all motivated to participate [63].

##### Opportunity for mid-level leaders and implementation leads: allocating administrative support

Two studies emphasized the importance of “adequate staffing and institutional support for patient recruitment [65].” The authors pointed out that identifying “patients was time consuming [69],” and thus required input from office staff and assistants. Depending on the clinical context, the individual making the decision to allocate staff time to supporting GMVs might be a mid-level leader or an implementation lead.

##### Innovation deliverers: capability and motivation

Twelve studies described the capabilities of the innovation deliverers as being essential to the success of the group visit programs. Some manuscripts emphasized the cultural expertise of the innovation deliverer, such as speaking the native language of innovation recipients [40] or sharing demographic characteristics with the recipients [54, 66] Others emphasized the skill and training of the innovation deliverers [48, 51, 52, 54, 66, 69, 70]. Some emphasized the importance of innovation deliverers being able to “step back while providing care in group visits [66]” and recipients described that “they came down on our level [44].”

Motivation, or ‘buy-in’ was also mentioned in two manuscripts as a particularly salient factor for innovation deliverers who provided integrative GMVs [58, 67]. The “openness” and “commitment” of innovation deliverers (clinicians delivering GMVs) to provide this unique type of care facilitated implementation.

#### Process

##### Assessing the needs of innovation recipients and innovation deliverers

Two studies discussed an assessment of the eligibility of patients to participate in the innovation, as well as the process of deciding what the eligibility criteria to participate in the GMVs should be [67, 69]. Some of the decisions over the inclusion criteria were based on “individual clinicians’ comfort with mental health conditions [67]” and providers’ assessment of which patients were suited to participating in groups.

##### Teaming, assessing context, and planning

One article discussed the importance of including administrative billing staff in the process of planning the implementation of a GMV program, particularly as it related to billing and financing of the program [55].

## Discussion

While only five of the studies included in this systematic review [38, 58, 65, 68, 69] explicitly set out to evaluate barriers and facilitators to implementation of GMVs for patients with chronic pain, the studies included point to consistent implementation determinants for this healthcare innovation. The relative advantage of GMVs for chronic pain when compared with other available models for treating chronic pain was mentioned in almost half of the manuscripts included in this review. Other commonly mentioned determinants included the capability and motivation of individual innovation deliverers (clinicians), the cost of the innovation to recipients and the health system including reductions in healthcare utilization, the need and opportunity of innovation recipients (patients), the availability of resources and any previous culture supporting groups within the inner setting (clinic), the relational connections supporting recruitment and referral to group visits within the inner setting, and financing and policies within the outer setting. Some less commonly mentioned determinants included policies within the outer setting related to reimbursement of complementary and integrative health practitioners, the pressures of the opioid crisis both within the outer setting and subsequent tension for change within the inner setting, the motivation of innovation recipients, the adaptability and design of the innovation for differing populations, opportunity for implementation leads to allocate administrative support, and the process of assessing needs, assessing context, teaming, and planning. Collectively, the determinants point to substantial opportunities related to the ongoing opioid and chronic pain epidemics and need for non-opioid treatment options, as well as specific challenges related to implementing GMVs for chronic pain.

The overall quality of manuscripts included in this review as assessed using the Mixed Methods Appraisal Tool was mixed, with the highest quality ratings obtained by qualitative studies. Although the focus of this systematic review was not on quantitative outcomes, it is noteworthy that there was a range of risk of bias in the manuscripts included, including incomplete data reported, and few randomized controlled trials. This suggests an opportunity for more rigorously designed controlled trials to be conducted on GMVs for chronic pain.

Of note, the participants in the studies included in this review included a high proportion of Black or African American participants and Hispanic participants. Some of the studies focused specifically on clinical settings serving underserved or minority populations, which is consistent with the use of GMVs as a strategy to promote health equity.

Our review is the first to focus on GMVs for chronic pain, and to include substantial data on barriers to broader implementation of GMVs. Recent systematic reviews of GMVs have assessed program components and barriers and facilitators of GMVs for chronic conditions [27, 30, 31], the use of GMVs for buprenorphine therapy [72], and GMVs for women’s health conditions [28]. A systematic review of patient-centered experience in GMVs/shared medical appointments for a wide range of conditions found many benefits to GMVs over individual care, including extended time, higher levels of patient satisfaction overall and with patient-clinician relationships, benefits of peer support, and high levels of engagement among patient participants [73]. Recently, a systematic review evaluating the potential of GMVs to address the Triple Aim of healthcare improvement found evidence of benefits of GMVs in all three aims [29].

While there is significant need from patients with chronic pain and healthcare organizations looking to implement guideline-concordant pain management, this review suggests that there remains a need for further study of determinants of GMV implementation for chronic pain. Our findings suggest that factors in the inner setting and the motivation of key decision-makers have a substantive impact on implementation. Environments with previous experience with GMVs, where leaders and innovation deliverers are motivated and have buy-in, and where referral and recruitment networks have been activated, are primary drivers of implementation. Similarly, the needs and opportunities of patients with chronic pain (such as access to transportation, technology, available time, or other chronic conditions that may impact group attendance) may impact implementation of groups. In communities where there is limited opportunity to attend group visits, including particularly acute transportation or technology barriers, attendance at group meetings may be difficult. However, the needs of patients and the relative advantages of GMVs may help to overcome barriers to attendance. Recent studies of GMVs for patients with chronic conditions conducted during the COVID-19 pandemic have found that conducting GMVs via telehealth is feasible and may have benefits for patients with chronic conditions, particularly to avoid contracting respiratory illnesses [74, 75].

The cost and financing of implementing GMVs are key implementation determinants, but there have been few evaluations of the cost of implementing GMVs or evaluations of financing policies to date. Research evaluating the potential for GMVs to reduce emergency department utilization suggests potential cost savings to health systems. Future evaluation of the cost effectiveness of GMVs for chronic pain as well as changes to financing and policies relevant to the implementation of GMVs (such as licensing of CIH professionals or including GMVs in bundled payments) could help to address some major barriers to implementation.

### Limitations

The implementation of GMVs for chronic pain is a topic that has generally been under-researched. With only five manuscripts explicitly focused on evaluating implementation determinants, this systematic review may be missing substantial context. Though other manuscripts included mention of implementation determinants in both their results and through contextual information incorporated into the body of manuscripts, these findings are limited in that these studies were not specifically designed to look at implementation issues.

Further, the majority of manuscripts included in this review are about studies conducted in the US. It is possible that there are alternative terms used in countries outside the US to describe comparable interventions to GMVs that the authors were not aware of. In the US, GMVs are often used as a strategy to overcome reimbursement barriers to guideline concordant chronic pain care. Although not conclusive, this may point to disproportionate use of GMVs for chronic pain care within the US context.

### Innovation

Although several systematic reviews have been conducted related to GMVs, this is the first systematic review to look specifically at implementation determinants for chronic pain GMVs. Use of the updated CFIR may enhance the ability to generalize and compare the findings presented here to other evaluations of implementation determinants. With thorough understanding of implementation determinants, there is potential to develop implementation strategies and increase access to GMVs, to understand if and how GMVs meet the Quintuple Aim for healthcare improvement [17].

## Conclusion

Group medical visits represent a potential innovation to improve access to guideline-concordant care for patients with chronic pain. There is urgency to implementing these innovations in the context of the ongoing opioid, chronic pain, and lingering COVID-19 pandemics. This review suggests that key determinants of implementation include the relative advantage of GMVs over other forms of chronic pain care, the motivation and capability of clinicians who will deliver GMVs, and the cost of GMVs to the healthcare system. Future research is needed to develop and test implementation strategies that address these determinants to promote the scale-up of GMVs for patients with chronic pain.

### Supplementary Information

Supplementary Material 1.

Supplementary Material 2.

## Data Availability

The datasets used and analyzed during the current study are available from the corresponding author on reasonable request.
