# Supplementary material for: Determinants of implementation for group medical visits for patients with chronic pain: a systematic review
Source: Implement Sci Commun. 2024 May 23;5:59. doi: 10.1186/s43058-024-00595-8 (PMC11112917; doi:10.1186/s43058-024-00595-8)
Supplement: Supplementary file 1 — Supplementary Material 1. [file 43058_2024_595_MOESM1_ESM.docx]

# Appendix A: Complete Search Strategy

Pubmed: ("Chronic Pain"[Mesh] OR “chronic pain” OR “chronic pains” OR "Fibromyalgia"[Mesh] OR fibromyalgia*[tiab] OR "Diabetic Neuropathies"[Mesh] OR neuropath*[tiab] OR "Low Back Pain"[Mesh] OR "low back pain" OR “lower back pain” OR "Headache Disorders"[Mesh] OR headache*[tiab] OR migraine*[tiab] OR "Anemia, Sickle Cell"[Mesh] OR “sickle cell anemia” OR “sickle cell anemias” OR “sickle cell disease” OR arthriti*[tiab] OR "Arthritis"[Mesh] OR “neurogenic pain” OR “psychogenic pain” OR “neuropathic pain” OR “nociceptive pain” OR “Visceral pain” OR “somatic pain”) AND **(shared medical appointments[mesh] OR "group medical visit" OR "group medical visits" OR "group medical appointment" OR "group medical appointments" OR "shared medical visit" OR "shared medical visits" OR "shared medical appointment" OR "shared medical appointments" OR "group visit" OR "group visits" OR "group appointment" OR "group appointments" OR "shared visit" OR "shared visits" OR shared appointment OR "shared appointments" OR ((shar*[ti] OR group*[ti]) AND (appointment*[ti] OR visit*[ti])))**

EMBASE: ('shared medical appointment'/exp OR 'shared medical appointment*' OR 'group medical visit'/exp OR 'group medical visit*' OR 'group visit*' OR 'medical group visit' OR 'group medical appointment*' OR 'shared medical visit*' OR 'group appointment*' OR 'shared visit*' OR 'shared appointment*' OR 'group pain management') AND ('chronic pain'/exp OR 'chronic pain' OR 'fibromyalgia'/exp OR fibromyalgia OR 'low back pain'/exp OR 'low back pain' OR 'migraine'/exp OR migraine* OR 'headache'/exp OR headache* OR 'neuropathy'/exp OR neuropath* OR 'sickle cell anemia'/exp OR 'sickle cell anemia' OR 'sickle cell disease')

Web of Science: ("shared medical appointment*" OR “group medical visit*" OR “group visit*" OR "medical group visit" OR "group medical appointment*" OR "shared medical visit*" OR "group appointment*" OR "shared visit*" OR "shared appointment*" OR "group pain management") AND ("chronic pain*" OR fibromyalgi*OR “low back pain*” OR migraine* OR headache* OR neuropath* OR "sickle cell”)

Cochrane Library: pain AND ("shared medical appointment*" OR "shared medical visit*" OR "group visit*" OR "group medical visits"
