# Supplementary material for: Determinants of implementation for group medical visits for patients with chronic pain: a systematic review
Source: Implement Sci Commun. 2024 May 23;5:59. doi: 10.1186/s43058-024-00595-8 (PMC11112917; doi:10.1186/s43058-024-00595-8)
Supplement: Supplementary file 2 — Supplementary Material 2. [file 43058_2024_595_MOESM2_ESM.docx]

| **Table 5a: CFIR 2.0 Domain: Innovation** | | | | | | | | | | | | | |
| --- | --- | --- | --- | --- | --- | --- | --- | --- | --- | --- | --- | --- | --- |
| **Determinants** | | **Data Source** | | | | | **Quotes** | | | | | **Context in Text** | |
| Relative advantage | | Cornelio-Flores 2018  pg. 130 | | | | | “Preference of the group visit versus the traditional doctor–patient encounter focused on two main aspects: perception of having more time with your doctor and the collaborative relation-ship among participants to learn new skills or knowledge about different health topics.” | | | | | Results, focus group data with patients | |
|  |  | Lestoquoy, 2017  p.36 | | | | | “the group as a space free of judgment, where participants do not feel defined solely by their health condition” (pg. 36) | | | | | Results, focus group data with patients | |
|  |  | Mehl-Madrona 2016  pg. 625 | | | | | “The physician co-leader reported enjoying seeing the patients in the 2 h GMV more than the same number of separate 15 min appointments. From his point of view, psycho-education about pain was delivered in a more effective and persuasive manner, and time existed to incorporate CAM therapies, which was not possible in 15 min individual appointments” | | | | | Discussion | |
|  |  | Meriwether 2022, p.697 | | | | | “Participants…wanted to build connections with  others who had ICBPS, and many saw it as a way of learning how to manage their ICBPS through tips from others. Patients also liked that Centering was not a traditional medical treatment, and appreciated its flexibility, where they could explore if they liked it and quit if they did not. | | | | | Results, qualitative focus group data with patients | |
|  |  | Rayburn 2017  (pg. 11) | | | | | “I only want to say thanks for helping us, because the exercises helped and everything else helped as well. I will recommend [the program] to my friends…More power to [the doctor and nurse] for a job well done. I am grateful and a big thank you to both of them. Very good communication and very friendly. More [programs] like this will be appreciated very much.” | | | | | Results, patient survey data | |
|  |  | Romanelli 2017  (pg. 150). | | | | | The [shared medical appointment] SMA model has several advantages over other types of group encounters, in that it is integrated with patients’ routine care and includes components of one-on-one visits. For this SMA, in particular, patients are referred by their treating provider, and pertinent information can be communicated through the EHR to these providers by the physician who facilitates the SMA, thereby promoting care coordination.” | | | | | Discussion | |
|  |  | Seesing, 2015  pg. 623 | | | | | “They provide patients an opportunity to share their knowledge and experiences as well as more time with their clinician, they may be more rewarding for both the patient and health professionals involved” | | | | | Discussion | |
|  |  | Shojania 2010  (pg. 628) | | | | | “The group visit model afforded the doctor more time to provide advice on various health issues and eliminated the need to repeat common advice... it was more satisfying to see the participants in a group setting because it allowed for more interesting interactions. These social interactions were powerful as participants received information from others with the same disease and benefited from finding answers to questions that they otherwise might not have thought to ask” | | | | | Discussion | |
|  |  | Spelman, 2017  pg. 2327 | | | | | “all patients indicated they would recommend the group visit to others and uniformly provided positive feedback regarding its utility” | | | | | Results, patient survey data | |
|  |  | Taube, 2021 | | | | | "reported positive feedback, stating that they were able to better understand their patients in addition to an improvement in communication amongst the team." | | | | | Discussion | |
|  |  | Thompson-Lastad 2018  pg. 1070 | | | | | “In many group visits, clinicians shared information about their own lives that they said they would not be as open about in standard care. For example, in most groups I observed, providers included themselves in group “check-ins,” discussing their own health goals alongside their patients” | | | | | Results, ethnographic observations | |
|  |  | Thompson-Lastad 2019  (pg. 5). | | | | | “IGMVs [Integrative Group Medical Visits] allowed patients to share expertise and support one another, which several described as patient-empowering. Providers’ favorite aspects of IGMVs included positive changes in patient–provider relationships” | | | | | Results, survey of clinicians and staff | |
|  |  | Wile 2021, p.5 | | | | | “interest in participation…sharing with others” | | | | | Results, interview with patients | |
|  |  | Wong 2015 pg. 36 | | | | | “The GMV structure also helped to neutralize the inherent power imbalance between patient and provider. GMVs were more interactive, allowing patients to gain information from their providers but also to listen and share their day-to-day management strategies with each other” | | | | | Results section, qualitative interviews with clinicians and patients | |
| Cost to patient and health system | | Geller, 2015  pg. 31 | | | | | “We continue to have these programs today and run them in a financially self-sustaining way. There is little extra financing, so group programs are limited to twice a week.” | | | | | Discussion | |
|  |  | Mehl-Madrona, 2016  pg. 624 | | | | | “GMVs averaged 12 people per session over 2 h. In this setting, the average income was $18 per person for a 2h group. This generated on average $204 per meeting or $408per month. Salaries for group leaders came to $320, which gave a net gain to the practice of $88 per month, which one hopes would cover overheads. Thus, improved care at a minimum broke even financially” | | | | | Results, cost data | |
|  |  | Seesing, 2015  pg. 622 | | | | | “group visits can be more cost-effective than individual visits provided that [shared medical appointment] SMA groups comprise more than 6 patients and that 75% of the patients are seen by their treating neurologist” | | | | | Results, cost data | |
|  |  | Smith, 2016  p.285 | | | | | “the capacity to overbook groups reduced the impact of participant non-attendance” | | | | | Discussion | |
| Design; Adaptability: Population served | | Meriwether 2022, p.696 | | | | | "Participants mentioned they thought having diversity within the Centering group was beneficial, particularly regarding age. Diversity, in their view, engendered a sense of comfort, lack of judgment, and a belief that ICBPS could affect anybody of any background" | | | | | Results, qualitative focus group data with patients | |
|  |  | Miller 2004(pg. 223). | | | | | “it may be more challenging to provide group care in a mixed ethnicity setting and that additional effort is needed to develop interventions that provide group care across cultural boundaries” | | | | | Discussion | |
| **Table 5b: CFIR 2.0 Domain: Inner Setting** | | | | | | | | | | | | | |
| **Determinants** | **Data Source** | | | | | **Quotes** | | | | | **Context in Text** | | |
| Tension for change: Alternative treatment options | Moitra, 2011  pg. 157 | | | | | “The development of clinic policies for opioid prescribing and increased consistency across prescribers were described as increasing cohesiveness among staff” | | | | | Results; Qualitative assessment with staff | | |
|  | Thompson-Lastad, 2020  pg. 2 | | | | | “Through our analysis of IMGVs for chronic pain management, we found that uncertainties surrounding the treatment of pain in the midst of the opioid crisis created similar space for the deployment of emerging forms of clinical practice, including IMGVs for chronic pain” | | | | | Introduction | | |
| Relational connections; Communications; Culture: Referral Systems | Gardiner 2019 | | | | | “our study team observed improved recruitment when study staff reflects the population being recruited, both in sex and in race and ethnicity. A [Patient Advisory Group] PAG is also very important in ensuring that all materials were at the right level of health literacy, and encouraged us to educate patients as what participation in a clinical trial means, for example providing more information on potential to benefit from participation and the definition of Integrative Medicine” (pg. 219).  “Providers could also refer patients through direct referral or “warm hand-offs” at clinics.” (pg. 217). | | | | | Discussion, Methods | | |
|  | Geller 2015  p. 29 | | | | | “Participants were recruited to the group medical visit program through physician referral by their primary care providers at [clinic] using the standard health center referral process. Providers were made aware of the groups by an email that was sent to all clinicians at our clinic. Referrals were handled on a first-come, first-served basis. When a referral was received, patients were screened for inclusion criteria and then invited to the group space for intake.” | | | | | Methods | | |
|  | Miller 2004  pg. 222 | | | | | “The higher response rate at site B (23%) is likely explained by the fact that patients at site B were selected by a primary provider and possibly encouraged by that provider to participate in the intervention” | | | | | Discussion | | |
|  | Moitra, 2011  pg. 157 | | | | | “Recruitment of patients to Pain Day was very successful, as most referring physicians were pleased and appreciative of offers to assist them in patient care” | | | | | Discussion | | |
|  | Roth 2021  p. S-75 | | | | | “Provider Willingness to refer patients to IMGV  Lack of process to schedule multiple patients at once  Lack of simple electronic referral process” | | | | | Results, qualitative interviews with clinicians, administrators, and staff | | |
|  | Spelman 2017  pg. 2328 | | | | | “~~In~~creasing eligible patient attendance in the intervention group was challenging, requiring modifications that targeted both clinicians and patients such as list distribution and pro-active e-mail reminders to clinicians, as well as a letter to patients informing them about the group.” | | | | | Discussion | | |
|  | Thompson-Lastad, 2019 | | | | | “respondents emphasized the need for adequate staffing and institutional support for patient recruitment, such as staff to make reminder phone calls to patients and to open facilities during evening hours when more patients are available.” ( pg. 5) | | | | | Results, survey of clinicians and staff | | |
| Available resources:  Previous culture supporting groups | Cornelio-Flores 2018  pg. 126 | | | | | “This intervention adapted the program developed by Dr. Gardiner at Boston Medical Center (BMC) for Latino Spanish speakers... Dr. Gardiner also supervised the adaptation of the CIM practices, and MBSR exercises and concepts in the curriculum.” | | | | | Methods | | |
|  | Gardiner 2014  pg. 21 | | | | | “All IMGV sessions took place in a conference room within the Family Medicine Ambulatory Care Clinic, a primary care outpatient clinic located in the hospital (cooking classes took place in the hospital’s teaching kitchen).” | | | | | Methods | | |
|  | Geller 2015  pg. 29 | | | | | “The chronic pain groups were modeled and designed similarly to the empowerment group visit model already being used at [clinic] for the treatment of obesity and diabetes” | | | | | Methods | | |
|  | Roth 2021  p.S-75 | | | | | “Stakeholders lack of familiarity with IMGV” | | | | | Results, qualitative interviews with clinicians, administrators, and staff | | |
|  | Spelman, 2017  p.2328 | | | | | “designing and implementing the group visit within our practice setting required relatively little time and effort; an average of 22.5 minutes of physician time was spent per patient attending the group, in addition to physician and health technician coordination time. This low investment was likely due to the fact that other group visits for conditions such as diabetes and opioid/chronic pain education have been implemented at our site.” | | | | | Discussion | | |
|  | Thompson-Lastad 2018  pg. 1067 | | | | | “The four organizations were chosen for their robust group medical visit programs; all have offered group visits for at least 10 years. Most began by offering the Centering Pregnancy model of group prenatal care or group visits for diabetes care, and have since expanded to offer a variety of group visits including some that incorporate complementary health approaches such as yoga, acupuncture, and meditation” | | | | | Methods | | |
| **Table 5c: CFIR 2.0 Domain: Outer Setting** | | | | | | | | | | | | | |
| **Determinants** | | | | | **Data Source** | | | | | **Quotes** | **Context in Text** | | |
| Financing; Policies and laws | | | | | Moitra 2011  (pg. 158). | | | | | “The level of complexity of the pain visit met the requirements of a consultation billing code with relatively higher reimbursement than typical PCP visits. However, Medicare discontinued consultation reimbursement part way through the program, resulting in lower payment for these complex visits. Reimbursement for the psychological service (i.e., group therapy) was 11% of charges billed and was insufficient to cover professional time” | Discussion | | |
|  |  |  |  |  | Rayburn 2017  pg. 12 | | | | | “Our institution has successfully billed for the program, reimbursed in the same way as a regular clinic visit” | Discussion | | |
|  |  |  |  |  | Thompson-Lastad 2019  pg. 5 | | | | | ‘‘How to serve patients with high co-pays.’’  ‘‘How to bill, who can bill.’’  ‘‘Is there a limit to how often [patients] can come and be billed for [group visits]?’’  “Specific challenges of IGMVs included finding and paying staff trained in integrative care given the lack of reimbursement for complementary health approaches” | Results, survey of clinicians and staff | | |
|  |  |  |  |  | Thompson-Lastad 2020  (pg. 258) | | | | | “The absence of frequent, long-term CIH treatment despite clinician and patient interest was a direct result of the fact that Medicaid and Medicare did not generally reimburse for CIH, even approaches that were recommended by major medical organizations” | Results section, qualitative interviews with clinicians and staff | | |
|  |  |  |  |  | Wile 2021  p.7 | | | | | “Participants had concerns about the cost of GMVs as well as the cost of related treatments” | Discussion | | |
|  |  |  |  |  | Wong 2015  pg. 37 | | | | | “Structural challenge for fee-for-service physicians was the financial risk of seeing fewer patients. This challenge was overcome by trying to ensure that between 10 and 15 patients were seen during a group visit” | Results section, qualitative interviews with clinicians and staff | | |
| Critical incidents and External pressure: Opioid Crisis and COVID-19 Pandemic | | | | | Mehl-Madrona 2016  pg. 623 | | | | | “Forty-one people who joined the GMV program did not reach the six-month mark. Three moved out of the area, 11 found other physicians who would prescribe opiates without restrictions, and 26 left because they failed to maintain their pain contract and were being tapered off opiates and found other care” | Results, administrative data | | |
|  |  |  |  |  | Moitra 2011  p.158 | | | | | “Many new patients presenting to the primary care clinic for pain treatment were expecting opioid prescriptions without assessment or monitoring. Many of these new clinic patients participated reluctantly or declined to participate in the GMV when they learned of clinic policies and procedures" | Discussion | | |
|  |  |  |  |  | Roth 2021, p.S-77 | | | | | “Because interviews were conducted during the COVID-19 pandemic, implementation strategies were designed to be conducted either in person or virtually (with some activities specified as telehealth-only).” | Results, qualitative interviews with clinicians and staff | | |
|  |  |  |  |  | Taube 2021, p.276 | | | | | “For patient and staff safety, non-essential face to face appointments were halted due to  COVID-19.” | Discussion | | |
| Policies and Laws: Licensing/credentialing of CIH practitioners | | | | | Thompson-Lastad 2019  pg. 6 | | | | | “Providers also had specific questions about staffing IGMVs with appropriately trained clinicians and support staff, and implementing and billing for complementary health approaches” | Results, survey of clinicians and staff | | |
|  |  |  |  |  | Thompson-Lastad 2020  (pg. 5). | | | | | **“**These varied staffing configurations depended primarily on the training of existing staff members, who typically played multiple roles at their workplaces (e.g. a licensed massage therapist who primarily worked as a coordinator of clinical programs, a licensed acupuncturist who primarily worked in an administrative role). In many cases, staff provided CIH exclusively in group visits because the lack of reimbursement meant it was not feasible to offer in individual visits” | Results section, qualitative interviews with clinicians and staff | | |
|  |  |  |  |  | Roth 2021  p. S-75 | | | | | “Clinical facilitator buy-in to deliver IMGV  Cofacilitator availability to conduct IMGV during clinical hours, Nursing staff not trained in check-in, Clinical facilitator's knowledge of IMGV eligibility,  Cofacilitator's preparation to lead IMGVs” | Results, qualitative interviews with clinicians, administrators, and staff | | |
| **Table 5d: CFIR 2.0 Domain: Individuals** | | | | | | | | | | | | | |
| **Determinants** | | | | **Data Source** | | | | | **Quotes** | | **Context in Text** | | |
| Innovation recipients: opportunity | | | | Gardiner, 2014  p.22 | | | | | ” In order to increase attendance and teach the principles of healthy eating, after each weekly meeting, the coordinator served a healthy buffet of food” | | Results | | |
|  |  |  |  | Gardiner 2019, Explore, p.219 | | | | | “The main reasons for declining was scheduling concerns (e.g. work during the day, too much of a time commitment,  n = 66), personal preferences about groups/ social contact (not wanting to be part of a group or not liking interacting with others, n = 33), Other reasons for declining included: medical concerns (surgeries scheduled, trouble with mobility, too many appointments/other medical commitments, n = 10),...childcare related problems (n = 8), transportation barriers (n = 10), and not speaking English as a first language (n = 2)” | | Results, interview data with patients | | |
|  |  |  |  | Meriwether, 2022 | | | | | "Participants expressed that most barriers to joining Centering were logistical, such as not being able to make the meeting time, use Zoom, or attend in person… although the convenience of doing Centering over Zoom was a positive aspect, meeting in person would have added more depth to their interactions and allowed them to connect socially with their peers…Some mentioned that notions about Centering, such as the belief that it was similar to a ‘support group’ were a barrier due to the stigma of grief and negativity around that term" | | Results, qualitative focus group data with patients | | |
|  |  |  |  | Miller 2004  pg. 222 | | | | | “Barriers to participation were similar to those mentioned in previously published research on group health interventions in socioeconomically disadvantaged populations and included problems with transportation, work conflicts, forgetting the appointment, family obligations, and complaints about insufficient personal attention” | | Discussion | | |
|  |  |  |  | Roth 2021  p.75 | | | | | “patients lack of familiarity with virtual platform” | | Results, qualitative interview data with staff, clinicians, and administrators | | |
|  |  |  |  | Shojania 2010  (pg. 628) | | | | | “Of the five patients who declined participation, two had conflicting work schedules, one had a conflicting babysitting schedule, one decided it was too great of a time commitment, and one had concerns regarding privacy” (pg. 626).  “Written suggestions for future programs indicated that, while it was beneficial to have sessions once per month, having the sessions on a particular day of the week caused some schedule conflicts” | | Results, survey data with patients | | |
|  |  |  |  | Spelman 2017  p. 2328 | | | | | “Increasing eligible patient attendance in the intervention group was challenging, requiring modifications that targeted both clinicians and patients such as list distribution and proactive e-mail reminders to clinicians, as well as a letter to patients informing them about the group.” | | Discussion | | |
|  |  |  |  | Taube 2021 | | | | | "some Veterans only attended two sessions" | | Discussion | | |
|  |  |  |  | Thompson-Lastad, 2019  (pg. 6) | | | | | “When patients are ill or they have transportation or health challenges and they miss a visit, it affects the whole group and the group dynamics” | | Results, survey of clinicians and staff | | |
|  |  |  |  | Wile, 2021 | | | | | “concerns about privacy and feeling vulnerable in a group” | | Results, interviews with patients | | |
| Innovation Recipients; Need | | | | Chao 2015, p.7 | | | | | “I enjoyed meeting other women with the same medical issues as me. It’s the first time I’ve met other women with the same problem. | | Results, interviews with patients | | |
|  |  |  |  | Gardiner, 2014  pg. 24 | | | | | “For the qualitative interviews, responses to open ended questions about patient satisfaction were positive overall. General themes included the impact IMGV had on their confidence and skill to self-manage pain. Qualitative data suggest that increased self-efficacy and improvement in symptoms (eg, mood, sleep disorders) may help mediate improvement in pain level” | | Results, qualitative interviews with patients | | |
|  |  |  |  | Harpole, 2003  pg. 723 | | | | | “Improvement was demonstrated not only by less headache days with less pain and less headache-related disability, but also by improved functional health status and increased satisfaction with headache care” | | Discussion | | |
|  |  |  |  | Huan, 2020  (pg. 88). | | | | | “Our findings suggest that the THRIVE program is effective at improving self-reported mental health (depression, anxiety, psycho- logical inflexibility, and experiential avoidance), life satisfaction, and how much pain interferes with work” | | Discussion | | |
|  |  |  |  | Romanelli, 2017  (pg. 148). | | | | | “Patients reported on average, improved confidence in self-managing pain and in their health care providers’ ability to help them manage their pain” | | Results, patient survey data | | |
|  |  |  |  | Shojania, 2010  (pg. 627). | | | | | “Comments about the sessions were all positive and appeared to fall within three categories: the participants benefited from communicating with other [chronic pain] patients, had constructive interactions with the doctor and visiting health professionals, and found the sessions informative/useful” | | Results, patient survey data | | |
|  |  |  |  | Smith, 2016  p.286 | | | | | “Many people perceive group interventions to be second-rate” | | Discussion | | |
|  |  |  |  | Taube, 2021 | | | | | "reported positive feedback, stating that they were able to better understand their patients in addition to an improvement in communication amongst the team." | | Discussion | | |
|  |  |  |  | Thompson-Lastad 2019  (pg. 5). | | | | | “Providers noted how IGMVs allowed patients to share expertise and support one another, which several described as patient-empowering. Providers’ favorite aspects of IGMVs included positive changes in patient–provider relationships. They also noted improvements in patients’ physical and mental health, which they attributed to both complementary health approaches and peer support” | | Results, survey of clinicians and staff | | |
|  |  |  |  | Wile 2021, p.5 | | | | | “pain impacting daily life… pain impact on mental health” | | Results, interviews with patients | | |
|  |  |  |  | Znidarsic 2021  pg. 188 | | | | | “Improvements in multiple domains of PROMIS-57 in our patients occurred in spite of the trend toward decreased use of opioid medications | | Discussion | | |
| Implementation deliverers: Community/context expertise | | | | Comelio-Flores, 2018  pg. 130 | | | | | “When asked about their thoughts on the intervention being delivered completely in Spanish, everyone expressed their gratitude for being able to communicate and interact with physicians and peers in their native language” | | Results, qualitative interviews with patients | | |
|  |  |  |  | Geller, 2015  pg. 31 | | | | | “It is the skills of group facilitation and management that become more important than the ultimate curriculum. Therefore, the reproducibility of these results relies on many factors that could be difficult to accurately measure, including facilitator skill” | | Discussion | | |
|  |  |  |  | Lestoquoy, 2017  pg. 37 | | | | | “the group changed when physicians participate: “They were our equals, they weren’t physicians, you know...They taught. ...They came down on our level and...When we do the exercise, they do the exercise.” | | Results, focus group data with patients | | |
|  |  |  |  | Mehl-Madrona, 2016  pg. 624 | | | | | “The leaders continually provided a gentle but consistent and persistent message that ‘‘you can learn to influence your pain.’’ Participants appeared to absorb this message over time.” | | Results | | |
|  |  |  |  | Meriwether, 2022 | | | | | ”Participants discussed that the group leader was beneficial to their Centering experience in that she had a calming presence, was able to unite people, and provided guidance for important aspects of the Centering group (eg, meditation)... While they saw health care providers as somewhat interchangeable or uniform in what they offer, they viewed the Centering facilitator as being unique and irreplaceable." | | Results, qualitative focus group data from patients | | |
|  |  |  |  | Miller, 2004  p.223 | | | | | “All the providers were trained in group facilitation techniques, and it is likely that their ability to address individual issues within a group context may have compensated for limited private contact with each participant…A provider who is approachable, engaged in the group process, comfortable delivering culturally competent care, and trained in group facilitation appears to be an integral part of building a successful GMV program…a number of the study participants expressed the concept of becoming friends with the GMV provider... the fact that the family physicians and nurse practitioners were women and good listeners may have contributed to the perception that they were a friend.” | | Discussion | | |
|  |  |  |  | Roth 2021  p.S-75 | | | | | “Clinical facilitator buy-in to deliver IMGV” | | Results, qualitative interviews with clinicians, administrators, and staff | | |
|  |  |  |  | Thompson-Lastad, 2018  (pg. 1070-1071). | | | | | “Practicing this way required a kind of retraining. When asked where they learned to step back while providing care in group visits, clinicians mentioned observing peers who practiced in groups visits or attending trainings with the Centering Health Care Institute, one of few organizations that provide formal training in group visits”  “Patients in some groups described support staff (such as medical assistants or health educators) as “group members.” These support staff were more often people of color than clinicians, as were the majority of patients” | | Results, ethnographic observations and interview data | | |
|  |  |  |  | Thompson-Lastad, 2020 | | | | | “IGMV programs were made possible in part because both patients and clinicians were open to CIH, an openness stemming in part from growing concern about opioid safety” (pg. 6).  “We found several conditions evident at all sites that made it possible to implement  IGMVs for chronic pain in safety-net clinics, the most notable being the presence of clinicians with particular commitments to IGMVs for pain management” (pg. 6). | | Results section, qualitative interviews with clinicians and staff | | |
|  |  |  |  | Wong, 2015  (pg. 38). | | | | | “Whether the facilitator was a provider or other staff, he/she was important in creating a safe environment where patients could comfortably share their experience, managing group dynamics, keeping the group relatively focused and ensuring delivery of specific medical services (e.g., review blood test results)” | | Results section, qualitative interviews with clinicians and staff | | |
|  |  |  |  | Znidarsic 2021  (pg. 184). | | | | | “Check-in was a time for the holistic psychotherapist to listen deeply to the language of the participants and assist with cognitive restructuring and reframing of negative thought patterns” | | Methods | | |
| Innovation recipients: motivation | | | | Meriwether 2022 | | | | | "They thought people should only pay for medical advice from a health care provider and not support from peers."  " For example, the qualitative study illuminated for investigators and clinicians that billing for the Centering visits was something that not only upset patients but might be a barrier to entry or diversity of the group." | | results, qualitative focus group data from participants | | |
|  |  |  |  | Spelman, 2017  p.2328 | | | | | “At these group visits, patients were engaged, valued the experience, and all requested prescriptions for the naloxone kit, suggesting the small out-of-pocket cost for veterans was not a barrier.” | | Discussion | | |
| **Table 5e: CFIR 2.0 Domain: Process** | | | | | | | | | | | | | |
| **Determinants** | | | **Data Source** | | | | | **Quotes** | | | | | **Context in Text** |
| Assessing needs | | | Thompson-Lastad 2020  pg. 258 | | | | | “Stratification was most visible in limited access to IGMVs for non-English speakers and people with severe mental health conditions, with notable exceptions… Individual clinicians' comfort with mental health conditions shaped which patients were welcomed into IGMVs” | | | | | Results, qualitative interview data with clinicians and staff |
|  |  |  | Wong 2015  pg. 37 | | | | | “Providers also identified patients whom they felt were less suited to participating in GMVs, including those who were hard of hearing, had limited English-speaking skills or cognitive deficits or were uncomfortable in groups” | | | | | Results, qualitative interview data with clinicians |
| Teaming, Assessing Context, Planning | | | Moitra 2011  pg. 158 | | | | | “Involve support staff, including administrative and billing staff, in planning because some ideas may not be feasible under managed care or with certain insurance payors.” | | | | | Discussion |
